# TLR4-NOX2 axis regulates the phagocytosis and killing of *Mycobacterium tuberculosis* by macrophages

**DOI:** 10.1186/s12890-017-0517-0

**Published:** 2017-12-12

**Authors:** Jingzhu Lv, Xiaoyan He, Hongtao Wang, Zhaohua Wang, Gabriel T. Kelly, Xiaojing Wang, Yin Chen, Ting Wang, Zhongqing Qian

**Affiliations:** 1 Department of Biochemistry and Molecular Biology, Bengbu Medical College, Bengbu, Anhui 233003 China; 2 Key Laboratory of Anhui Province for Infection and Immunology, Bengbu Medical College, 2600 Donghai Ave, Bengbu, Anhui 233003 China; 3 Department of Pulmonary Medicine, Bengbu Infectious Disease Hospital, Bengbu, Anhui 233003 China; 4 Department of Medicine, The University of Arizona, 1656 E. Mabel St, P.O. Box 245218, Tucson, AZ 85724 USA; 5 Anhui Clinical and Preclinical Key Laboratory of Respiratory Disease, Department of Respiration, First Affiliated Hospital; Bengbu Medical College, Bengbu, Anhui 233000 China; 6 Department of Pharmacology and Toxicology, The University of Arizona, Tucson, AZ 85724 USA

**Keywords:** TB, TLR, NOX2, Phagocytosis

## Abstract

**Background:**

Macrophages stand at the forefront of both innate and adapted immunity through their capacities to recognize, engulf, and eliminate foreign particles, and to stimulate adapted immune cells. They are also involved in controlling pro- and anti-inflammatory pathways. Macrophage activity against *Mycobacterium tuberculosis* (*M. tuberculosis*) has been shown to involve Toll-like receptor (TLR) activation and ROS production. Previous studies have shown that lipopolysaccharide (LPS), through TLR4, could activate macrophages, improve their bactericidal ROS production, and facilitate anti-infective immune responses. We sought to better understand the role of the TLR4-NOX2 axis in macrophage activation during *M. tuberculosis* infection.

**Methods:**

THP-1 macrophages and PMA primed THP-1 macrophages [THP-1(A)] were treated with LPS and infected by *M. tuberculosis*. Cells were analyzed by flow cytometry for TLR4 expression, ROS production, phagocytosis, and killing of *M. tuberculosis*. Western blotting was used to analyze NOX2 expression. Inhibitors of the TLR4-NOX2 pathway were used to assess this pathway’s role in these processes, and their role in LPS activation of macrophages.

**Results:**

We found that THP1-derived macrophages or PMA primed THP-1 macrophages exhibit higher surface TLR4 levels and increased NOX2 expression levels following LPS treatment. *M. tuberculosis* infection reduced these levels, but LPS was able to limit the negative effects of *M.tb*. Additionally, LPS increases THP-1(A) cells’ bactericidal activities including phagocytosis, ROS production, and destruction of *M. tuberculosis.* Significantly, all of these activities are impaired when TLR4 or NOX2 are inhibited.

**Conclusion:**

These studies demonstrate the importance of the TLR4-NOX2 axis in *M. tuberculosis* elimination by macrophages and may lead to novel therapies for tuberculosis and other bacterial infections.

**Electronic supplementary material:**

The online version of this article (10.1186/s12890-017-0517-0) contains supplementary material, which is available to authorized users.

## Background

Macrophages, a key host-defense cell type, recognize invading pathogens via pathogen-associated pattern recognition receptors to initiate anti-infection innate immune responses [1]. During *Mycobacterium tuberculosis* (*M. tuberculosis*) infection, Toll-like receptors (TLRs) on the surface of macrophages are shown to recognize pathogens, trigger endocytosis to form phagosomes, produce cytokines, improve cell apoptosis, and thus exert bactericidal activities [2]. The activation of macrophages is the first step of acquiring specific anti-TB immunity, which inhibits the growth or even kills *M. tuberculosis*, and thus is an important defense mechanism in controlling the infection and spread of *M. tuberculosis* [3]. TLR4, similar to some other TLR family members [4], is a critical molecule in the anti-TB immune response, through its ability to recognize *M. tuberculosis* and its components to trigger further innate immune responses [5].

Upon recognition and phagocytosis of *M. tuberculosis*, macrophages produce excessive reactive oxygen species (ROS) and other bactericidal substances to kill and inactivate *M. tuberculosis* [6]. Previous studies have shown that lipopolysaccharide (LPS) could activate macrophages through TLR4 to improve the production of ROS, thus directly exerting bactericidal activities, and facilitating the anti-infective immune response [7]. NADPH oxidase (NOX) may produce superoxide ions and ROS through the aggregation and activation of oligomeric enzymes to participate in the host immune defense [8], and reduced NOX activation may increase the risk of infection in patients [9]. In addition, TLR signaling pathways could affect the synthesis and activation of NOX (mainly NOX2), and thus regulate the level of inflammation [10–12]. These findings strongly suggest a possible central role for the TLR-NOX-ROS signaling axis in host defense against *M. tuberculosis* by macrophages.

In this study, we aim to confirm the role of the TLR4-NOX2 axis in the phagocytic and bactericidal functions of macrophages in *M. tuberculosis* infection. The characterization of this key pathway in macrophage-mediated innate immunity against *M. tuberculosis* will reveal details relating to the molecular mechanism of *M. tuberculosis* infection, and facilitate the identification of novel therapies.

## Methods

### Cells and bacteria

The human acute monocytic leukemia cell line THP-1 was purchased from American Type Culture Collection (ATCC, Manassas, USA), suspended in Roswell Park Memorial Institute (RPMI) medium 1640 (Sigma-Aldrich, Shanghai, China) to achieve a density of 5 × 10^5^/mL, and cultured in a six-well culture plate. Then, 2 mL of complete culture medium was added to each well. Phorbol-12-myristate-13-acetate (PMA, 120 ng/mL, Sigma-Aldrich, Shanghai, China) was added to stimulate the cells for 24 h, which induced the macrophage-like change in the cells [THP-1(A)] [13]. An attenuated strain of *Mycobacterium tuberculosis* (H37Ra) was purchased from the Type Culture Collection of Chinese National Institute (Beijing, China) for the Control of Pharmaceutical and Biological Products (batch number: 9,302,025). The H37Ra strain was seeded in solid medium and incubated at 37 °C in a constant-temperature incubator. When the cells reached the logarithmic phase (about 1 month), an aseptic inoculating loop was used to transfer a small amount of bacterial inoculum into a 1-mL homogenizer containing some normal saline. The mixture was homogenized repeatedly, and normal saline was used to wash the concentrate twice (10,000 rpm, 2 min each). Then, a 1-mL syringe was used to add 0.9% NaCl to the bacterial suspension, which was pipetted repeatedly to obtain a single bacterial suspension. The concentration of the bacteria was measured with a spectrophotometer at 600 nm. The concentration of the bacteria was adjusted to 5 × 10^8^/mL, and the suspension was stored in an Eppendorf (EP) tube at 4 °C till use.

### PBMC collection

All protocols of the clinical study involving human participants have been approved by the ethics committee of Bengbu Medical College and signed informed consent was obtained from all participants. 20 mL acid-citrate dextrose-treated blood was obtained from healthy donors or TB patients. PBMCs were collected by centrifugation over lymphocyte separation medium, and washed three times with phosphate-buffered saline.

### Antibody, blocker, and probe

PMA (#P1585) and lipopolysaccharide were obtained from Sigma-Aldrich (Shanghai, China), fluorescein-labeled antibody TLR4-PE from eBioscience (San Diego, USA), anti-human TLR4 blocking antibody HTA125 and anti-human NOX2 protein antibody from Santa Cruz Biotechnology (Santa Cruz, USA), and NOX2 inhibitor diphenyleneiodonium (DPI) from Sigma. Fluorescein isothiocyanate (FITC) and dimethyl sulfoxide were from Merck (Kenilworth, USA). Fluorescein diacetate (FDA) was from Invitrogen (New York, USA).

### Grouping and treatment of the cells

The bacteria were used to infect the cells at a ratio of 1:10, and the TLR4 blocking antibody HTA125 (1 μg/mL) or the NOX2 inhibitor DPI (20 μM) was used to pretreat the infected and noninfected cells, after which LPS (100 ng/mL, Sigma-Aldrich, Shanghai, China) was used to stimulate the cells for 24 h. Cells not treated with the blocker were also used in the study as the control.

### Measuring NOX2 protein expression level by western blotting

After treatment, the cells were treated with cell lysis buffer to extract the total protein. After electrophoresis, transfer, incubation with primary and secondary antibodies, and enhancement with 3,3′-diaminobenzidine development, a Tanon GIS gel documentation system was used to obtain and record the images. Imaging analysis software was used for the quantitative analysis of the grayscale of the bands.

### Measuring TLR4 expression and ROS levels using flow cytometry

The cells in each group were collected into the tubes of the flow cytometer and washed, for a final volume of 30 μL/tube. Fluorescent probe DHR123 (10 μL/tube, purchased from Sigma-Aldrich) was added to the control group, while 30 μL/tube unlabeled *M. tuberculosis* suspension (5 × 10^8^/mL) and 10 μL/tube DHR123 (final concentration of 1 μmol/L) were added to the experiment tubes, mixed, and incubated in a 37 °C water bath for 15 min. Then, the cells were quickly washed in ice-cold phosphate-buffered saline (PBS) twice, and flow cytometry was used to measure the ROS level in the *M. tuberculosis*-infected THP-1(A) cells. For each sample, the level in 10,000 cells was measured. The region of THP-1(A) was first selected in the forward scatter and side scatter two-dimensional scatter diagram, and then a FL1(DHO) histogram was used to analyze the mean fluorescence intensity (MFI) of the oxidized rhodamine123 (namely DHO), which represented the cellular ROS level. Blank control group (macrophage group) and macrophage + DHR123 15-min group were also used in the measurement.

### Measuring TLR4 and NOX2 mRNA expression levels using fluorescence quantitative polymerase chain reaction

TRIzol was used to extract the total RNA from the cells. The sequences of TLR4 primers were as follows: F: ACCTGTCCCTGAACCCTATGAA, R: CTTCTAAACCAGCCAGACCTTG. The sequences of NOX2 primers were as follows: F: CAAGATGCGTGGAAACTACC, R: TTGAGAATGGATGCGAAGG. The sequences of human glyceraldehyde-3-phosphate dehydrogenase (GAPDH) primers were as follows: F: TGCACCACCAACTGCTTAGC, R: GGCATGGACTGTGGTCATGAG. The amplification was performed for 40 cycles. After completion of the amplification, the amplification curve and solubility curve of the real-time polymerase chain reaction were obtained, and the 2^-**ΔΔ**CT^ method was used to analyze the expression of *TLR4* and *NOX2* mRNA in the cells.

### Measuring the phagocytosis and killing activity of THP-1(A) on *M. tuberculosis*

THP-1(A) cells derived from THP-1 after induction with 120 ng/mL of PMA were obtained and transferred into the tubes of a flow cytometer (1 × 10^5^/tube). Then, 10 μL/tube of FITC-labeled *M. tuberculosis* suspension (5 × 10^6^/mL), or FDA-labeled *M. tuberculosis* suspension (5 × 10^8^/mL) was added, mixed, incubated in a 37 °C water bath for 30 min, and then quickly washed with ice-cold PBS twice (10,000 rpm, 2 min each). The supernatant was discarded, and the cells were resuspended. Complete RPMI-1640 culture medium containing 10% fetal bovine serum (FBS) was added (100 μL/tube), the cells were incubated in a 37 °C water bath, and 200 μL/tube ice-cold tri-distilled water was added to each tube at time 0, 10, 30, or 60 min. The tubes were incubated for 2 min to lyse the cells, and then flow cytometry was used to obtain the time-phagocytosis kinetic curve of THP-1(A) against FITC-labeled *M. tuberculosis,* and time-killing kinetic curve of THP-1(A) against FDA-labeled *M. tuberculosis*.

THP-1(A) cells were seeded on a six-well plate (2 mL/well). For the cells in the control group, complete RPMI-1640 culture medium was added. However, for the cells in the LPS treatment group, different concentrations of LPS (final concentrations of 10, 100, 1000, and 10,000 ng/mL) were added to stimulate the cells for 24 h. Ice-cold PBS was used to elute the cells into the tubes of the flow cytometer, and 100 μL of complete culture medium was used to resuspend the cells. Nonlabeled bacterial suspension (10 μL/tube) was added to the blank control group, while 10 μL/tube FITC-labeled or FDA-labeled *M. tuberculosis* (labeling rate > 90%) was added to the negative control and LPS treatment groups. After incubation in a 37 °C water bath for 30 min, the tubes were quickly washed in ice-cold PBS (10,000 rpm for 5 min) twice. The supernatant was discarded, and 100 μL/tube of complete RPMI-1640 culture medium was added to each tube. Then, the tubes were incubated in 0 °C or 37 °C water baths for 0−60 min, and ice-cold tri-distilled water (250 μL/tube) was added immediately. After incubation for 2 min, flow cytometry was used to measure the MFI of FITC or FDA in *M. tuberculosis*.

The TLR4 and NOX2 blocker groups were also used in this study. In brief, the cells were pretreated with HTA125 (1 μg/mL) for 30 min or DPI (10−20 μM) for 90 min. Then, LPS (100 ng/mL) was used to stimulate the cells for 24 h, and the cells were eluted into the corresponding flow cytometer tubes with ice-cold PBS. The cells were incubated with 5 × 10^6^/mL *M. tuberculosis*-FITC or 5 × 10^8^/mL *M. tuberculosis*-FDA for 30 min, washed in ice-cold PBS, and then washed again with ice-cold RPMI-1640 medium once. Next, complete culture medium containing 10% FBS was added, and the tubes were incubated in a 37 °C bath for 0−60 min. Ice-cold tri-distilled water (200 μL/tube) was added to lyse the cells for 2 min, and flow cytometry was used to measure the killing rate. THP-1(A) added to the unlabeled bacteria was used as the blank control.

### Statistical analysis

SPSS 17.0 for Windows software (SPSS, IL, USA) was used for the statistical analysis. All data is expressed as mean and standard deviation ($\overline{x}\pm s$) and compared by one-way analysis of variance or *Q* test. A *P* value less than 0.05 was considered statistically significant.

## Results

### LPS upregulates TLR4/NOX2 expression and ROS levels in THP-1 cells

**Fig. 1:**
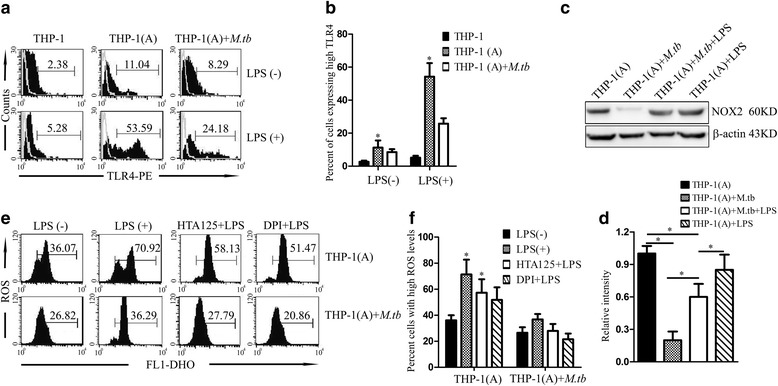
LPS upregulates TLR4 and NOX2 expression and ROS levels in THP-1 cells. **a** THP-1 cells or THP-1(A) cells were treated with or without LPS, incubated with TLR4-PE, and bound TLR4-PE levels were measured using flow cytometry as an indicator of TLR4 expression. Additionally, some THP-1(A) cells were infected with *M. tuberculosis*. Histograms of the results, with percentage of cells expressing high TLR4 indicated. **b** Bar graph of percentages from **a**. (*; *P* < 0.05 compared to its control). **c** Western blot of NOX2 expression in THP-1(A) cells with or without pretreatment with LPS, with or without subsequent *M. tuberculosis* infection. **d** Bar graph of band intensities from **c**. (*; *P* < 0.05). **e** Intracellular ROS levels were measured using flow cytometry. **f** Bar graph of ROS levels from **e**. [*; *P* < 0.05 compared to LPS(−)]

As a surface marker of leukocytes, TLR4 was quantified with flow cytometry methods [14]. LPS challenge (24 h) significantly increased TLR4 expression on naïve THP-1 cells or PMA-activated THP-1 cells [THP-1(A) cell] (Fig. 1a, b). Interestingly, PMA treatment and LPS challenge exert a synergistic effect on TLR4 upregulation. Similarly, LPS significantly increased surface TLR4 expression on *M. tuberculosis*-infected THP-1(A) cells (Fig. 1a, b). We next examined the expression of NOX2 protein under similar conditions. *M. tuberculosis*-infected THP-1(A) cells exhibit reduced NOX2 protein expression, compared to naïve THP-1(A) cells. Since NOX2 is known to be responsible for immune-defensive ROS generation [15], *M. tuberculosis* infection seems to reduce NOX2 expression to suppress the immune response in macrophages. However, LPS challenge (100 ng/mL, 24 h) significantly promoted NOX2 protein expression in *M. tuberculosis-*infected THP-1(A) cells (Fig. 1c, d). In addition, we have confirmed that the regulation of TLR4 and NOX2 expression is on the mRNA levels (Additional file 1: Figure S1).

NOX2 exerts its immune-defensive effects via ROS generation upon pathogen stimulation [16]. Thus we next examined the intracellular ROS levels in THP-1(A) cells. The ROS level in the *M. tuberculosis-*infected THP-1(A) cells was significantly lower than that in the uninfected cells, consistent with our findings on NOX2 expression regulation in these cells. In addition, LPS stimulation (24 h) significantly promoted ROS generation in infected cells (Fig. 1e, f). To confirm the regulation of ROS generation is dependent on TLR4-NOX2 axis, the selective TLR4 inhibitor HTA125 and the NOX2 inhibitor DPI were used. HTA125 or DPI treatment significantly reduced the ROS level in *M. tuberculosis-*infected THP-1(A) cells post LPS stimulation (Fig. 1e, f). These findings confirm that generation of immune-defensive ROS is dependent on the TLR4-NOX2 axis.

### Blockade of TLR4 and NOX2 inhibits phagocytosis of *M. tuberculosis* by THP-1 cells

**Fig. 2:**
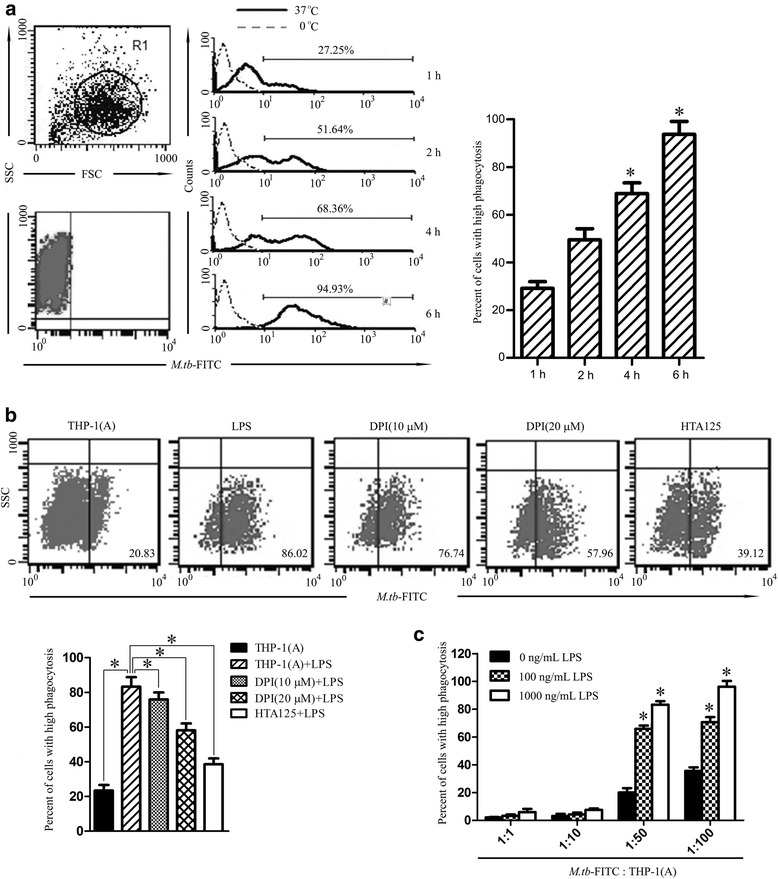
Blocking TLR4 and NOX2 inhibits the phagocytosis of *M. tuberculosis* by THP-1 cells. **a** THP-1(A) cells were incubated with *M. tuberculosis*-FITC for various time periods (1–6 h) at 0 °C or 37 °C. THP-1(A) cells were isolated and identified by flow cytometry (top left), and amount of *M. tuberculosis*-FITC in those cells was also measured by flow cytometry (bottom left). Histogram of results (middle) with percentage of cells containing high levels of FITC indicated for the 37 °C group. Bar graph of percentages (right). (*; *P* < 0.05 compared to *t* = 1 h). **b** THP-1(A) cells treated without LPS, with LPS, or with LPS in combination with DPI (10 μM), DPI (20 μM), or HTA125 pretreatment were infected with *M. tuberculosis*-FITC, isolated, and analyzed by flow cytometry. The proportion of cells containing high levels of FITC is indicated (top). Bar graph of percentages (bottom). (*; *P* < 0.05). **c** THP-1(A) cells were incubated with various concentrations of LPS (0–1000 ng/mL) and subsequently infected with various concentrations of *M. tuberculosis*-FITC (1:1–1:100). The proportion of cells containing high levels of FITC was measured as in **b**. Bar graph of the results. (*; *P* < 0.05 compared to lowest concentration of LPS). (FSC; forward scatter, SSC; side scatter)

After we confirmed that LPS restores the expression of TLR4 and NOX2 and generation of ROS in *M. tuberculosis* infected THP-1(A) cells, next we examined the effects of LPS on the phagocytic activity of THP-1(A) cells. THP-1(A) cells were incubated with FITC labeled *M. tuberculosis* (1:50) for different time periods (1, 2, 4, and 6 h) and the phagocytic effects of the cells were determined by flow cytometry methods. The mean fluorescence intensity (MFI) of FITC was detected and used as an indicator of phagocytic activity in THP-1(A) macrophages. *M. tuberculosis* infection increased phagocytosis of THP-1(A) time-dependently and peaked at 6 h (Fig. 2a). In addition, *M. tuberculosis* infection dose dependently activated the phagocytic effects of THP-1(A) cells (Fig. 2c). These findings also have been reported previously [17], proving a working model of phagocytosis assessment in the THP-1(A) system. As expected, LPS (100 or 1000 ng/mL 24 h pretreatment) significantly increased phagocytosis of *M. tuberculosis* (at the ratio of 1:50 and 1:100, 30 min) in THP-1(A) cells (Fig. 2c). We also found that LPS dose-dependently (10−10,000 ng/mL) activates phagocytosis in both THP-1 and THP-1(A) cells incubated with *M. tuberculosis*-FITC at the ratio of 1:50 for 30 min (Additional file 2: Figure S2).

We next tested the role of TLR4-NOX2 in LPS-activated phagocytosis in *M. tuberculosis* infected THP-1(A) cells. THP-1(A) cells were activated by LPS (1000 ng/mL) for 24 h and then incubated with *M. tuberculosis* at the ratio of 1:50 for 30 min. The phagocytosis rate of *M. tuberculosis* was significantly increased by LPS priming (Fig. 2b). However, the TLR4 receptor blocker HTA125 (1 μg/mL, 30 min pretreatment) significantly reduced phagocytosis in THP-1(A) cells treated by LPS and infected by *M. tuberculosis*. Similarly, NOX2 inhibitor DPI pre-treatment (10 μM, 90 min) significantly reduced the LPS stimulated *M. tuberculosis* phagocytosis in THP-1(A) cells, and a higher concentration of DPI (20 μM) further attenuated phagocytosis. These findings have demonstrated that LPS activates phagocytosis in *M. tuberculosis* infected cells via the TLR4-NOX2 axis.

### Blockade of TLR4-NOX2 inhibits the killing of *M. tuberculosis* by THP-1 cells

**Fig. 3:**
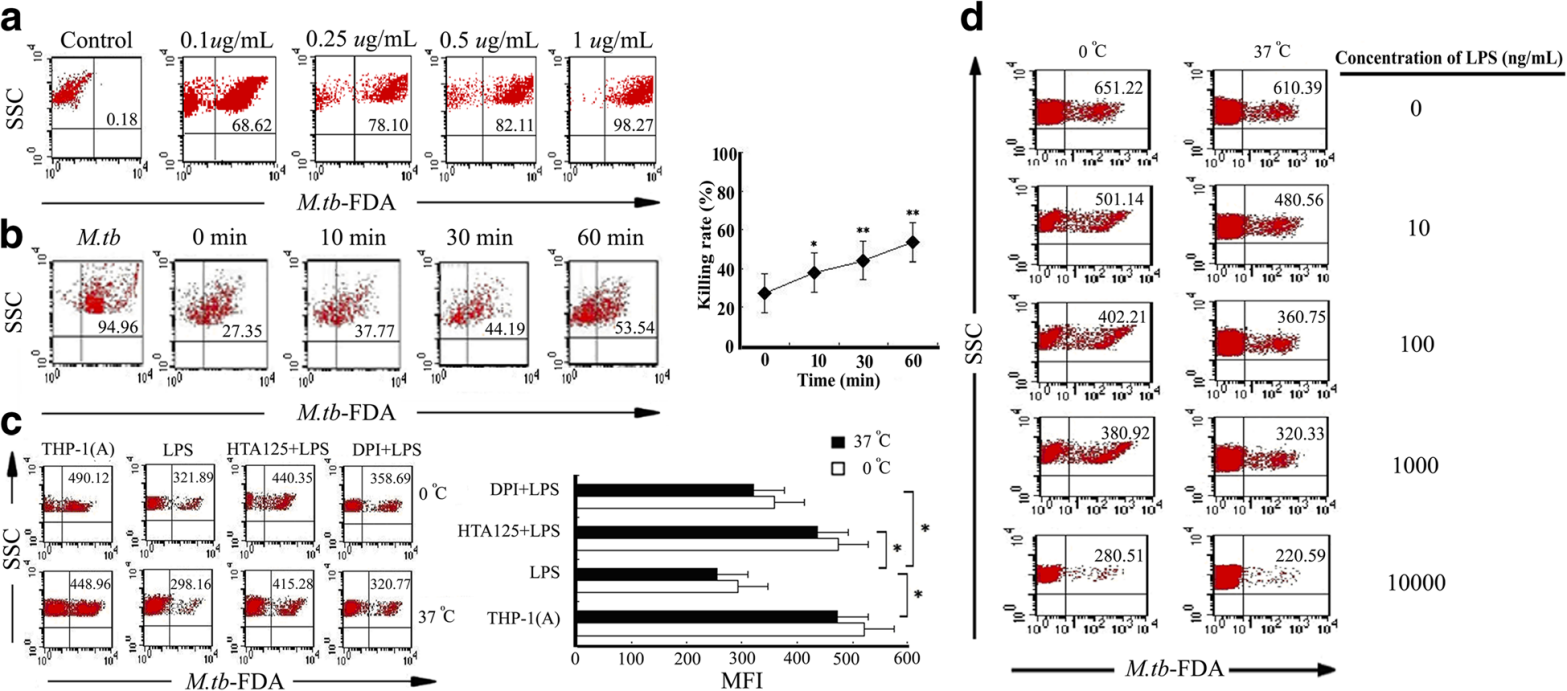
Blocking TLR4 and NOX2 inhibits the killing of *M. tuberculosis* by THP-1 cells. **a** *M. tuberculosis* was incubated with various concentrations of FDA to achieve labeling. Percentage of bacteria labeled is indicated. **b** FDA labeled *M. tuberculosis* (left most scatter plot) was incubated with THP-1(A) cells for various time periods (0–60 min). THP-1(A) cells were lysed and total phagocytized *M. tuberculosis* and *M. tuberculosis* in serum were isolated. FDA levels in *M. tuberculosis* were measured by flow cytometry with percentage of *M. tuberculosis* with FDA removed indicated (left). Graph of FDA removal levels (right). (*; *P* < 0.05 compared to t = 0, **; *P* < 0.01 compared to t = 0). **c** THP-1(A) cells treated without LPS, with LPS, or with LPS in combination with DPI or HTA125 pretreatment were infected with *M. tuberculosis*-FDA at 0 °C or 37 °C. THP-1(A) cells were lysed and total phagocytized *M. tuberculosis* and *M. tuberculosis* in serum were analyzed by flow cytometry for FDA levels. The number of viable *M. tuberculosis* retaining FDA is indicated (left). Graph of MFI from left (right). (*; *P* < 0.05). **d** THP-1(A) cells were treated with various concentrations of LPS (0–10,000 ng/mL), then infected with *M. tuberculosis*-FDA at 0 °C or 37 °C. THP-1(A) cells were lysed and total phagocytized *M. tuberculosis* and *M. tuberculosis* in serum were analyzed by flow cytometry for FDA levels. The number of viable *M. tuberculosis* retaining FDA is indicated. (SSC; side scatter, MFI; median fluorescence intensity)

We next examined the effect of LPS on bactericidal activity of THP-1(A) cells. We successfully labeled *M. tuberculosis* with FDA (0.1–1.0 μg/mL) and analyzed it by flow cytometry (Fig. 3a). The maximum labeling efficiency was achieved at an FDA concentration of 1.0 μg/mL (98.27% ± 0.92). The bactericidal effects of THP-1(A) on *M. tuberculosis*-FDA were found to be time dependent, with the proportion of FDA removal peaking at 60 min (53.54% ± 1.85) (Fig. 3b). The time point 30 min was chosen to evaluate the role of TLR4 and NOX2 on bactericidal activity of THP-1(A) cells. After pretreatment with the anti-TLR4 receptor antibody HTA125 (1 μg/mL), the number of viable *M. tuberculosis*-FDA was increased significantly at both 0 °C and 37 °C compared to LPS treatment alone (*P* < 0.05) (Fig. 3c). Similarly, the NOX2 inhibitor DPI significantly reduced bactericidal activity at both 0 °C and 37 °C compared to LPS treatment alone (*P* < 0.05). LPS was also found to increase bactericidal activity in THP-1(A) cells in a dose dependent manner, with significant effects observed at concentrations above 100 ng/mL at both 0 °C and 37 °C and continuously increasing until the maximum testing dose at 10,000 ng/mL (*P* < 0.01) (Fig. 3d).

## Discussion

Macrophages are one of the first lines of defense against bacteria invasion through their ability to directly recognize and eliminate microbes. LPS, a component of Gram-negative bacteria including *M. tuberculosis*, induces pro-inflammatory activities in various cell types [18]. Here, we demonstrate the role of LPS in stimulating bactericidal activities against *M. tuberculosis* by macrophages. We found that LPS increased surface levels of TLR4 and induced expression of NOX2. Consistently, we found that LPS enhanced ROS generation, phagocytosis, and bactericide activities in THP-1(A) macrophages. Significantly, all these activities induced by LPS were dependent on the TLR4-NOX2 axis. As an interesting validation, we used PBMCs isolated from TB patients or healthy volunteers, and examined the levels of TLR4 expression (Additional file 3: Figure S3), as well as intracellular ROS levels (Additional file 4: Figure S4). We confirmed that LPS restored reduced surface expression of TLR4, as well as intracellular ROS levels in the TB-infected PBMCs.

*M. tuberculosis* is mainly recognized by macrophages via TLR2/4 signaling pathways, but the TLR2 signal can be inhibited by the antigens secreted by bacteria [19, 20]. In addition, bacterial components such as phosphatidylinositol mannosides strongly inhibit TLR4 signaling. The combined effects of these factors effectively inhibit autophagy and allow the bacteria to survive for prolonged periods of time in macrophages [21, 22]. *M. tuberculosis* contains multiple TLR agonists (for instance, the TLR2 agonists LpqH, LprA, and LprG). However, such agonists cannot effectively activate TLR signaling and dowstream phagocytic and bactericidal functions [23]. The possible reasons are: (1) the TLR2 signal is inhibited by some antigens secreted by *M. tuberculosis* and (2) the bacterial components such as phosphatidylinositol mannosides strongly inhibit the TLR4 signal. The combined effects of these factors ultimately inhibit the effective activation of macrophages [24].

In this study, we showed that TLR4 expression in *M. tuberculosis*-infected macrophages was significantly decreased, suggesting that *M. tuberculosis* could downregulate TLR4 expression to inhibit and evade cognition by macrophages. Biglycans were shown to induce NOX2 expression in mouse macrophages through a TLR4/MyD88-dependent pathway, which also led to NOX2 activation [25]. The present study showed that, in addition to upregulating TLR4 expression, LPS could also enhance the bactericidal activity of macrophages by activating the TLR4-NOX2 axis to produce ROS. Previous studies have shown that TLR signaling on activated macrophages could enhance NOX2 activity and stimulate ROS, which not only directly damaged *M. tuberculosis* but also initiated the activation of autophagy. All these effects contribute to anti-*M. tuberculosis* responses [16, 26].

A previous study has shown that TLR’s could affect NOX2 expression, activate NOX2 to produce ROS, and regulate inflammatory responses [10]. This study showed that LPS could upregulate NOX2 expression and ROS production in *M. tuberculosis*-infected THP-1 cells through TLR4-dependent activation. This indicates that regulating ROS through TLR’s could be a potential target for anti-*M. tuberculosis* treatment.

As noted, we used *M.tb*-FITC as a tool to reflect the relative level of intracellular *M.tb*. Measurement was made through an immunofluorescence microtip sensor for specific detection of Mycobacterium cells in sputum samples by the combination of electric field, streaming flow, and immuno-affinity binding [27–29]. This assay is widely used to determine relative levels of *M.tb* levels. This method is relatively limited due to its low reliability, especially for those assays using heterogeneous cell samples. For the current study, this method is convenient, and the results are conclusive, especially combined with all other supportive data.

In summary, our work has merged several pathways related to bactericidal activities discovered in other immune system cells and we extended these ideas to THP-1 macrophages. We demonstrated *M. tuberculosis* elimination’s reliance on the TLR4-NOX2 axis. These findings add to our understanding of macrophage immune action, and may contribute to novel therapies for tuberculosis and other bacterial infections.

## Conclusions

LPS treatment of THP-1 macrophages leads to increased TLR4 and NOX2 expression, and enhances their ability to produce ROS, phagocytize, and kill bacteria such as *M. tuberculosis*. Each of these processes is dependent on the TLR4-NOX2 axis. This work demonstrates the ability for LPS to activate macrophages through the TLR4-NOX2 axis, the reliance of macrophages on the TLR4-NOX2 axis for its functions, and may contribute to developing therapies for bacterial infections.

## Additional files

Additional file 1: Figure S1. LPS upregulates TLR4 and NOX2 mRNA levels in *M. tuberculosis*-infected THP-1 cells. THP-1(A) cells were treated as in Fig. 1c. mRNA was isolated and the levels of TLR4 and NOX2 mRNA were measured, compared to GAPDH, and normalized to untreated. (*; *P* < 0.05). (TIFF 252 kb)

Additional file 2: Figure S2. LPS dose-dependently (10−10,000 ng/mL) activates phagocytosis in both THP-1 and THP-1(A) cells incubated with *M. tuberculosis*-FITC at the ratio of 1:50 for 30 min. THP-1 and THP-1(A) cells were incubated with various concentrations of LPS (0–10,000 ng/mL) and subsequently infected with *M. tuberculosis*-FITC, isolated (top scatter plots), and analyzed by flow cytometry (bottom scatter plots). The proportion of cells containing high levels of FITC is indicated. (TIFF 82 kb)

Additional file 3: Figure S3. LPS upregulates TLR4 expression in peripheral blood mononuclear cells (PBMCs) from TB patients. PBMCs were isolated from patients with active tuberculosis and healthy volunteers. Age and gender factors were considered to match. In the supplementary experiments, 5 × 10^5^/mL PBMCs from patient or health control were treated with or without 100 ng/mL LPS for 6 h, and TLR4 levels were measured using flow cytometry. The bar graph showed statistical difference of TLR4 levels (*; *P* < 0.05 compared to health control). (TIFF 265 kb)

Additional file 4: Figure S4. LPS upregulates ROS levels in peripheral blood mononuclear cells (PBMCs) from TB patients. PBMCs were isolated from patients with active tuberculosis and healthy volunteers. Age and gender factors were considered to match. In the supplementary experiments, 5 × 10^5^/mL PBMCs from patient or health control were treated with or without 100 ng/mL LPS for 6 h, and ROS levels were measured using flow cytometry. The bar graph showed statistical difference of ROS levels (*; *P* < 0.05 compared to health control). (TIFF 203 kb)

## Abbreviations

DPI: Diphenyleneiodonium
FDA: Fluorescein diacetate
FITC: Fluorescein isothiocyanate
FSC: Forward scatter
LPS: Lipopolysaccharide
MFI: Median fluorescence intensity
NOX: NADPH oxidase
PBS: Phosphate-buffered saline
PMA: Phorbol-12-myristate-13-acetate
ROS: Reactive oxygen species
RPMI: Roswell Park Memorial Institute medium 1640
SSC: Side scatter
THP-1(A): PMA-activated THP-1
TLR: Toll-like receptor
