# Supplementary figures and images for: TLR4-NOX2 axis regulates the phagocytosis and killing of Mycobacterium tuberculosis by macrophages
Source: BMC Pulm Med. 2017 Dec 12;17:194. doi: 10.1186/s12890-017-0517-0 (PMC5727946; doi:10.1186/s12890-017-0517-0)

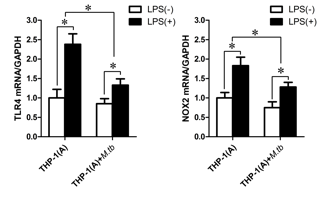

Supplement: Supplementary file 1 — LPS upregulates TLR4 and NOX2 mRNA levels in M. tuberculosis-infected THP-1 cells. THP-1(A) cells were treated as in Fig. 1c. mRNA was isolated and the levels of TLR4 and NOX2 mRNA were measured, compared to GAPDH, and normalized to untreated. (*; P < 0.05). (TIFF 252 kb) [file 12890_2017_517_MOESM1_ESM.tiff]

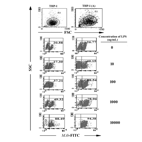

Supplement: Supplementary file 2 — LPS dose-dependently (10−10,000 ng/mL) activates phagocytosis in both THP-1 and THP-1(A) cells incubated with M. tuberculosis-FITC at the ratio of 1:50 for 30 min. THP-1 and THP-1(A) cells were incubated with various concentrations of LPS (0–10,000 ng/mL) and subsequently infected with M. tuberculosis-FITC, isolated (top scatter plots), and analyzed by flow cytometry (bottom scatter plots). The proportion of cells containing high levels of FITC is indicated. (TIFF 82 kb) [file 12890_2017_517_MOESM2_ESM.tiff]

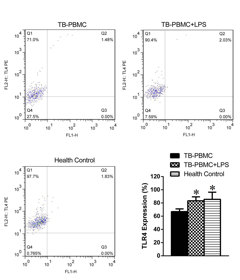

Supplement: Supplementary file 3 — LPS upregulates TLR4 expression in peripheral blood mononuclear cells (PBMCs) from TB patients. PBMCs were isolated from patients with active tuberculosis and healthy volunteers. Age and gender factors were considered to match. In the supplementary experiments, 5 × 10^5^/mL PBMCs from patient or health control were treated with or without 100 ng/mL LPS for 6 h, and TLR4 levels were measured using flow cytometry. The bar graph showed statistical difference of TLR4 levels (*; P < 0.05 compared to health control). (TIFF 265 kb) [file 12890_2017_517_MOESM3_ESM.tiff]

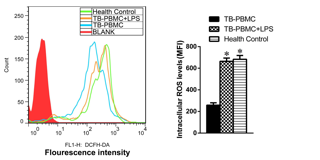

Supplement: Supplementary file 4 — LPS upregulates ROS levels in peripheral blood mononuclear cells (PBMCs) from TB patients. PBMCs were isolated from patients with active tuberculosis and healthy volunteers. Age and gender factors were considered to match. In the supplementary experiments, 5 × 10^5^/mL PBMCs from patient or health control were treated with or without 100 ng/mL LPS for 6 h, and ROS levels were measured using flow cytometry. The bar graph showed statistical difference of ROS levels (*; P < 0.05 compared to health control). (TIFF 203 kb) [file 12890_2017_517_MOESM4_ESM.tiff]
